# Thromboembolism, bleeding and vascular death in nonvalvular atrial fibrillation patients with type 2 diabetes receiving rivaroxaban or warfarin

**DOI:** 10.1186/s12933-021-01250-5

**Published:** 2021-02-26

**Authors:** Craig I. Coleman, Olivia S. Costa, Christopher W. Brescia, Burcu Vardar, Khaled Abdelgawwad, Nitesh Sood

**Affiliations:** 1 University of Connecticut School of Pharmacy, 69 North Eagleville Road, Unit 3092, Storrs, CT 06269 USA; 2 Evidence-Based Practice Center, Hartford Hospital, Hartford, CT USA; 3 Department of Data Science, Freshtech IT, LLC, East Hartford, CT USA; 4 Bayer AG, Berlin, Germany; 5 Arrhythmia Services, Southcoast Health, Fall River, MA USA

**Keywords:** Diabetes, Cardiology, Anticoagulant

## Abstract

**Background:**

Diabetes increases a patient’s risk of developing atrial fibrillation by 49%. Patients with nonvalvular atrial fibrillation are at a fivefold increased risk of stroke and die more frequently from vascular causes. We sought to evaluate the effectiveness and safety of rivaroxaban versus warfarin in nonvalvular atrial fibrillation patients with type 2 diabetes.

**Methods:**

This was an analysis of Optum® De-Identified electronic health record data from 11/2010 to 12/2019. We included adults with nonvalvular atrial fibrillation and type 2 diabetes, newly started on rivaroxaban or warfarin and with ≥ 12-months of prior electronic health record activity. Patients who were pregnant, had alternative indications for oral anticoagulation or valvular heart disease were excluded. We evaluated the incidence rate (%/year) of developing the composite outcome of stroke/systemic embolism or vascular death and major or clinically relevant nonmajor bleeding as well as each endpoint individually. Hazard ratios with 95% confidence intervals were calculated using propensity score-overlap weighted proportional hazards regression.

**Results:**

We included 32,078 rivaroxaban (31% initiated on 15 mg dose) and 83,971warfarin users (time-in-therapeutic range = 47 ± 28%). Rivaroxaban was associated with a reduced risk of stroke/systemic embolism or vascular death (3.79 vs. 4.19; hazard ratio = 0.91, 95% confdience interval = 0.88–0.95), driven mostly by reductions in vascular death (2.81 vs 3.18, hazard ratio = 0.90, 95% confidence interval = 0.86–0.95) and systemic embolism (0.13 vs. 0.16; hazard ratio = 0.82, 95% confidence interval = 0.66–1.02). Major/clinically relevant nonmajor bleeding was less frequent with rivaroxaban versus warfarin (2.17 vs. 2.31; hazard ratio = 0.94, 95% confidence interval = 0.89–0.99) due to decreased critical organ bleeding (including intracranial hemorrhage) (0.35 vs. 0.54; hazard ratio = 0.63, 95% confidence interval = 0.55–0.72).

**Conclusions:**

In nonvalvular atrial fibrillation patients with type 2 diabetes, rivaroxaban was associated with an ~ 10% relative reduction in vascular mortality and fewer bleeding-related hospitalizations versus warfarin.

## Background

Nonvalvular atrial fibrillation (NVAF) substantially increases patients’ risk of stroke by fivefold and mortality by twofold [1, 2]. Oral anticoagulation (OAC) with either a vitamin K antagonist (VKA) or a direct-acting oral anticoagulant (DOAC), such as apixaban, dabigatran, edoxaban or rivaroxaban, significantly decreases the risk of clot formation and subsequent morbidity and mortality in NVAF populations.

Diabetes, including type 2 diabetes (T2D) increases patients’ risk of developing NVAF by 49% [3, 4]. The prevalence of atrial fibrillation in diabetes, including type 2 diabetes (T2D) is twofold compared to those without diabetes. [1–3]. The presence of T2D in NVAF patients increases their risk of both stroke/systemic embolism (SSE) and vascular death compared with those without diabetes [3–6]. Data from randomized controlled trials (RCTs) [7, 8] and administrative claims database analyses [9] demonstrate rivaroxaban is at least as effective and safe as warfarin in preventing SSE in patients with NVAF and T2D. Similar analyses performed in electronic health record (EHR) datasets, which provide more nuanced clinical patient data, are scarce.

In this study, we sought to assess the incidence rates of SSE/vascular death and major or clinically relevant nonmajor (CRNM) bleeding complications in NVAF patients with comorbid T2D receiving either rivaroxaban or warfarin.

## Methods

We performed a cohort analysis within the US Optum® De-Identified EHR data set [10]. EHR data from November 1, 2010 through December 31, 2019 was utilized for this study. Rivaroxaban was approved for NVAF in the US in November 2011, and therefore, utilization of data back to November 2010 was required to provide a full 12-month pre-index period for all patients. The Optum EHR data set includes longitudinal patient-level medical record data for 91+ million patients seen at 700+ hospitals and 7000+ clinics across the US. This database contains data on insured and uninsured patients of all ages to provide a representative sample of US patients with NVAF. It includes records of prescriptions and over-the-counter medications (as prescribed or self-reported by patients), laboratory results, vital signs, anthropometrics, other clinical observations, diagnoses (ICD-9 and ICD-10) and procedures codes (ICD-9, ICD-10, CPT-4, HCPCS, Revenue codes). The use of the provided Optum® De-Identified EHR data was determined by the New England Institutional Review Board (IRB) to not constitute research involving human subjects and was therefore exempt from board oversight.

Adult patients (≥ 18 years-of-age) with NVAF and comorbid T2D, who were OAC-naïve, newly-initiated on rivaroxaban or warfarin after November 1, 2011 (defined as the index date), active in the data set for at least 12-months prior to the index date (based on the “First Month Active” field in the Optum data set) and with documented care in the EHR from at least one provider in the 12-months prior to the index date were eligible for study inclusion. Patients with valvular heart disease (defined as any rheumatic heart disease, mitral stenosis or mitral valve repair/replacement), any prior OAC use per written prescription or patient self-report during the 12-month pre-index period, receiving rivaroxaban doses other than 15 mg once daily or 20 mg once daily, had venous thromboembolism as an alternative indication for OAC use, underwent recent orthopedic knee or hip replacement, or who were pregnant were excluded. Given the high specificity (> 98%) of billing codes for identifying T2D, the presence of a code for T2D was considered sufficient to indicate its presence regardless of hemoglobin A1c value (which is also a treatment goal) [11]. Due to the moderate sensitivity of billing codes for detecting T2D (~ 60–70%) [11], patients without a billing code for diabetes but with a hemoglobin A1c > 6.5% and receiving a non-insulin antihyperglycemic medication were also considered to have T2D.

To adjust for potential confounding between the rivaroxaban and warfarin cohorts, we calculated propensity scores based upon multivariable logistic regression [12] which included commonly used variables and accepted risk factors for differential OAC exposure identified during the baseline period including demographics, comorbidities, laboratory and vital signs and concurrent outpatient co-medication use. Covariates included in the propensity score model are denoted in Table 1. The presence of a comorbid disease diagnoses was determined based upon billing codes and/or supporting laboratory/observation data. The absence of data suggesting a comorbidity exists was assumed to represent the absence of the disease (thus categorical covariates had no missing data). When dependence on billing codes was required to identify covariates, we utilized endorsed and/or validated coding algorithms (e.g., Centers for Medicare and Medicaid Services Chronic Conditions Warehouse, Elixhauser or Charlson comorbidity indices), whenever possible [13–16]. For continuous laboratory and observation variables with < 25% values missing, data were imputed using a multiple imputation approach based on a fully conditional specification linear regression model with all other available covariates and the outcomes of interest included in the model [17]. Estimated propensity scores were subsequently used to weight patients for analysis using an overlap weighting approach [18, 19]. Overlap weighting assigns weights to patients that are proportional to their probability of belonging to the opposing treatment cohort (i.e., rivaroxaban patients were weighted by the probability of receiving warfarin (or 1—the propensity score) and warfarin patients were weighted by the probability of receiving rivaroxaban (the propensity score). Overlap weighting was chosen as the primary method for confounder adjustment in this study because it allows for all eligible patients to be included in the analysis (unlike propensity score matching which typically results in sample size reduction in one or both cohorts), it assigns greater weight to patients in which treatment cannot be predicted and lesser weight to patients with extreme propensity scores (approaching 0.0 or 1.0) preventing outliers from dominating the analysis and decreasing precision [a concern with inverse probability weighting (IPTW)], and because overlap weighting has the favorable property of resulting in the exact balance (absolute standardized differences = 0) of all variables included in the multivariable logistic regression model used to derive the propensity score [12, 18, 19].

**Table 1:** Unweighted and weighted characteristics of included patients

	Unweighted			Propensity score overlap weighted		
	Rivaroxaban, % N = 32,078	Warfarin, % N = 83,971	ASD, %	Rivaroxaban, % N = 32,078	Warfarin, % N = 83,971	ASD, %
Demographics						
Age, years (mean ± SD)^a^	70 ± 10	73 ± 10	30.0	71 ± 10	71 ± 10	0.0
Age 65–74 years	34.2	31.5	6.8	33.8	33.8	0.0
Age ≥ 75 years	36.4	48.1	26.6	41.0	41.0	0.0
Female	39.9	40.8	2.1	40.5	40.5	0.0
White race, self-reported	85.6	86.8	5.6	86.4	86.4	0.0
Hospital frailty score, intermediate risk	37.3	39.0	4.0	38.1	38.1	0.0
Hospital frailty score, high risk	15.8	24.3	29.6	18.2	18.2	0.0
Hospitalizations in prior 12-months (mean ± SD)	0.98 ± 1.84	1.22 ± 1.98	12.4	1.05 ± 1.83	1.05 ± 1.83	0.0
Medical history						
Ablation	2.6	3.1	10.0	2.7	2.7	0.0
Active cancer	5.1	5.4	3.3	5.3	5.3	0.0
Active gastric or duodenal ulcer in prior 90-days	0.2	0.4	3.8	0.2	0.2	0.0
Acute coronary syndrome	10.4	13.2	14.9	11.2	11.2	0.0
Anxiety	15.0	14.2	3.5	14.8	14.8	0.0
Any bleeding in prior 90-days	3.0	5.0	29.3	3.5	3.5	0.0
Asthma	10.9	10.0	5.3	10.5	10.5	0.0
Hemoglobin A1c < 7%	52.1	54.7	5.8	52.9	52.9	0.0
Hemoglobin A1c 7–8%	23.3	22.8	1.6	23.0	23.0	0.0
Hemoglobin A1c > 8%	24.6	22.5	6.4	24.0	24.0	0.0
Body mass index 30–3 9.9 kg/m^2^	45.0	41.8	7.2	43.8	43.8	0.0
Body mass index ≥ 40 kg/m^2^ or body weight > 120 kg	26.3	22.7	10.8	25.1	25.1	0.0
Cardioversion	7.5	7.9	3.1	7.5	7.5	0.0
Carotid endarterectomy and/or stent	0.8	1.1	17.7	0.9	0.9	0.0
Chronic obstructive pulmonary disease	24.0	27.4	9.8	25.2	25.2	0.0
Coagulopathy	5.8	10.2	33.8	6.9	6.9	0.0
Crohns disease or ulcerative colitis	0.7	0.8	7.4	0.8	0.8	0.0
Chronic venous insufficiency	4.9	6.4	15.6	5.2	5.2	0.0
Dementia	4.9	7.2	22.6	5.7	5.7	0.0
Depression	17.1	17.9	3.1	17.4	17.4	0.0
Diverticular disease	6.5	7.1	5.2	6.7	6.7	0.0
eGFR 30–50 mL/min	9.5	13.9	23.7	11.2	11.2	0.0
eGFR < 30 mL/min	3.3	13.6	84.3	4.6	4.6	0.0
Kidney transplant or dialysis	0.8	7.2	124.8	1.2	1.2	0.0
Excessive alcohol consumption	0.8	0.8	0.0	0.8	0.8	0.0
Gastroesophageal reflux disease	25.3	25.7	1.2	25.5	25.5	0.0
Heart failure	33.6	45.8	28.3	37.3	37.3	0.0
Helicobacter pylori infection	0.3	0.3	0.0	0.3	0.3	0.0
Hemoglobin < 13 g/dL in men or < 12 g/dL in women (anemia)	40.5	57.6	38.1	45.8	45.8	0.0
Hypercoagulable state	0.5	0.8	26.1	0.6	0.6	0.0
Hyperlipidemia	82.7	80.6	7.7	82.2	82.2	0.0
Hypertension	91.3	90.2	7.2	90.8	90.8	0.0
Systolic blood pressure ≥ 160 mmHg	3.9	3.5	6.2	3.7	3.7	0.0
Diastolic blood pressure ≥ 100 mm Hg	5.0	3.0	29.3	4.1	4.1	0.0
Ischemic stroke	7.7	10.1	16.4	8.6	8.6	0.0
Ischemic stroke in prior 12 months	2.3	3.0	15.1	2.0	2.0	0.0
Liver dysfunction	5.6	7.3	15.6	6.0	6.0	0.0
Major bleed	1.2	2.7	45.6	1.5	1.5	0.0
Major adverse limb events	6.4	9.8	25.5	7.3	7.3	0.0
Major surgery in prior 90-days	40.6	44.6	9.0	41.8	41.8	0.0
Osteo- or rheumatoid arthritis	23.3	22.3	3.1	23.2	23.2	0.0
Osteoporosis	6.7	8.2	12.0	7.3	7.3	0.0
Pneumonia	11.4	15.5	19.6	12.6	12.6	0.0
Psychosis	2.0	2.9	21.0	2.2	2.2	0.0
Proteinuria	3.8	3.9	1.5	3.8	3.8	0.0
Revascularization (CABG or PCI)	20.8	26.3	16.9	22.7	22.7	0.0
Sleep apnea	24.7	22.4	7.1	23.6	23.6	0.0
Smoker	13.8	11.5	11.5	13.0	13.0	0.0
Vascular disease (prior MI, PAD or aortic plaque)	26.8	33.1	16.6	28.7	28.7	0.0
Body weight < 60 kg	3.7	5.2	19.6	4.2	4.2	0.0
Anti-hyperglycemic medications						
Dipeptidyl peptidase-4 inhibitor	11.5	9.3	13.1	10.7	10.7	0.0
Glucagon-like peptide-1 analog	4.9	2.4	40.8	3.7	3.7	0.0
Insulin	29.2	36.6	18.5	31.0	31.0	0.0
Metformin	51.5	38.6	28.9	47.8	47.8	0.0
Sodium-glucose cotransporter-2 inhibitor	3.4	1.0	68.8	2.2	2.2	0.0
Sulfonylurea or glinide	25.9	28.1	6.2	26.8	26.8	0.0
Thiazolidinediones	4.5	3.6	12.8	4.2	4.2	0.0
Other medications						
Amiodarone	11.8	15.4	17.0	13.1	13.1	0.0
ACE inhibitor or ARB	70.7	65.1	14.2	69.3	69.3	0.0
Alpha blocker	14.7	16.7	8.3	15.3	15.3	0.0
Aspirin	28.5	29.4	2.4	29.0	29.0	0.0
Barbiturate	1.2	1.2	0.0	1.3	1.3	0.0
Benzodiazepine	16.5	17.2	2.8	16.7	16.7	0.0
Beta blocker	73.2	74.0	2.3	73.3	73.3	0.0
Dihydropyridine calcium channel blocker	5.4	4.7	8.1	5.0	5.0	0.0
Digoxin	9.5	14.9	28.2	11.4	11.4	0.0
Diltiazem	20.0	17.7	8.3	19.3	19.3	0.0
Dronedarone	1.9	1.1	30.6	1.6	1.6	0.0
Estrogen	1.6	1.2	16.1	1.4	1.4	0.0
Histamine-2 receptor antagonist	9.3	11.0	10.3	9.8	9.8	0.0
Levothyroxine	16.7	18.6	7.2	17.3	17.3	0.0
Loop diuretic	38.1	52.0	31.2	43.0	43.0	0.0
Nonsteroidal anti-inflammatory drug	23.4	16.7	23.2	21.0	21.0	0.0
Other anti-arrhythmic agent	8.8	5.8	24.8	7.9	7.9	0.0
Other antidepressant	10.1	10.9	4.7	10.4	10.4	0.0
Other antiplatelet agent	1.3	1.3	0.0	1.3	1.3	0.0
Other cholesterol medication	13.6	13.5	0.5	13.5	13.5	0.0
P2Y12 inhibitor	6.9	7.0	0.9	6.9	6.9	0.0
Proton pump inhibitor	35.6	38.2	6.2	36.2	36.2	0.0
SSRI or SNRI	22.2	22.3	0.3	22.2	22.2	0.0
Statin	70.0	69.7	0.8	70.0	70.0	0.0
Thiazide diuretic	30.5	26.2	11.7	29.2	29.2	0.0
Verapamil	1.8	1.8	0.0	1.9	1.9	0.0
Time in therapeutic INR range (mean ± SD)^a^ Median (25%, 75%)	–	46 ± 28 47 (21, 66)	–	–	47 ± 28 50 (24, 69)	–
CHA_2_DS_2_VASc score (mean ± SD)^a^	4.2 ± 1.5	4.6 ± 1.5	–	4.3 ± 1.5	4.3 ± 1.5	–
CHADS_2_ score (mean ± SD)^a^	3.1 ± 1.2	3.4 ± 1.2	–	3.2 ± 1.2	3.2 ± 1.2	–
Modified HAS-BLED score (mean ± SD)^a^	1.5 ± 0.8	1.7 ± 0.9	–	1.5 ± 0.9	1.5 ± 0.8	–

*ASD* absolute standardized difference, *eGFR* estimated glomerular filtration rate, *INR* international normalized ratio, *SD* standard deviation, *SSRI* selective serotonin reuptake inhibitor, *SNRI* serotonin-norepinephrine reuptake inhibitor

^a^Covariate not included in the propensity score model

Our study’s co-primary outcomes included the incidence rates (%/year) of developing the composite of SSE/vascular death (effectiveness) and major/CRNM bleeding resulting in hospitalization (safety) [20]. Individual components of the composite outcomes were also assessed. Vascular death was defined as primary diagnosis/procedure code for acute coronary syndrome, venous thromboembolism, aortic plaque, carotid stenosis, carotid stenting, heart failure, hypertension, intracranial hemorrhage (ICH), ischemic heart disease, stroke, major adverse limb event, myocardial infarction, peripheral artery disease, systemic embolism, ventricular fibrillation/arrest or revascularization associated with a hospital admission or emergency room visit within 365 days of the date of death. Our major bleeding component was intended to approximate the International Society of Thrombosis and Hemostasis (ISTH) definition of major bleed, and was defined as an intracranial hemorrhage, critical organ per ISTH or other bleed associated with a fall in hemoglobin level of ≥ 2 g/dL or requiring transfusion of ≥ 2 units of whole blood or red cells [21]. Study outcomes were defined based on ICD-9/10-CM diagnosis codes, CPT-4, HCPCS, ICD-9/10-PCS procedure codes or laboratory, vital signs, and other patient observation results. We also performed falsification analysis using urinary tract infection as an outcome.

Baseline characteristics were analyzed using descriptive statistics. Categorical variables were reported as percentages and continuous variables as means ± standard deviations (SDs). Propensity score-overlap weighted Cox proportional hazards regression models using a robust estimator [15] were employed to calculate hazard ratios (HRs) with corresponding 95% confidence intervals (CIs) for all outcomes. The proportional hazard assumption was tested based on Schoenfeld residuals (and was found valid in all cases). Patients were censored in the Cox models at time of outcome occurrence, end-of-EHR activity (based on “Last Month Active” data available in the Optum EHR) or end-of-data availability (December 31, 2019).

Sensitivity analysis were performed whereby we assessed the SSE/vascular death and major/CRNM bleeding outcomes after applying stabilized IPTW and 1:1 propensity score matched (using a caliper of 0.25 standard deviations of the of the logit of the propensity score) approaches to confounding adjustment and capping the duration of patient follow-up at a maximum of 2-years. Subgroup analyses stratifying patients by age (≥ 80, < 80 years), sex, baseline estimated glomerular filtration rate (eGFR) (> 50, 30–50, < 30 mL/min/1.73m^2^), baseline hemoglobin A1c (≥ 8.5%, < 8.5%), the presence of absence of morbid obesity (defined as a body mass index (BMI) ≥ 40 kg/m^2^ or body weight > 120 kg), heart failure, vascular disease (defined as myocardial infarction, peripheral artery disease or aortic plaque), peripheral artery disease, cardiac revascularization within the prior 12-months (coronary artery bypass grafting or percutaneous coronary intervention), prior stroke, concomitant aspirin use (anytime during the 90-days after the index date), frailty (low, moderate-to-high Hospital Frailty scores) [16], rivaroxaban dose (initiated on 20 mg or 15 mg once daily) and warfarin time in therapeutic range (TTR) (< 25%, 25 to < 50%, 50 to < 75%, ≥ 75%). Propensity score models and overall weighting were re-run for each subgroup analysis including the same variables as the overall analysis.

All database management and statistical analysis were performed using SAS version 9.4 (SAS Institute, Cary, NC) and IBM SPSS version 27.0 (IBM Corp., Armonk, NY). A p-value < 0.05 was considered statistically significant in all cases. P-values for heterogeneity across subgroups were adjusted to control false discovery rates due to multiple testing [22].

The use of the Optum EHR data set was reviewed by the New England Institutional Review Board and was determined to be exempt from oversight, as this research project did not involve human subject research and the investigators were supplied only de-identified and HIPAA-compliant data [10].

This report was written to comply with the Reporting of Studies Conducted using Observational Routinely Collected Health Data for Pharmacoepidemiology (RECORD-PE) statement [23].

## Results

**Fig. 1:**
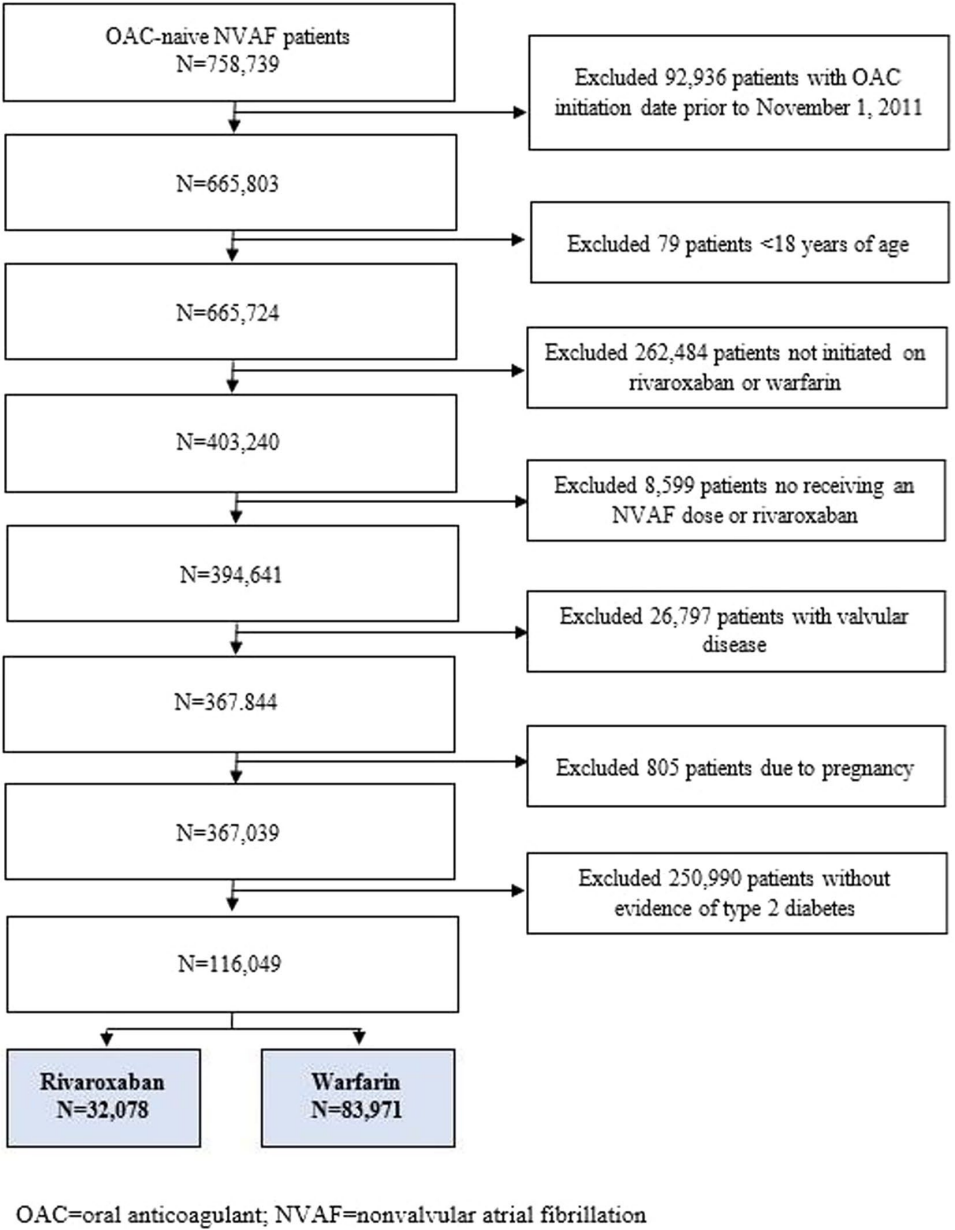
Selection of patients

We identified 32,078 rivaroxaban and 83,971 warfarin patients with NVAF and comorbid T2D (Fig. 1). Weighted and unweighted baseline characteristics of included patients are depicted in Table 1. After propensity score overlap weighting, the rivaroxaban and warfarin cohorts were identical (standardized difference = 0 for all). Of included patients, 99% had a diagnostic code for T2D. The average CHA_2_DS_2_VASc score was 4.3 ± 1.5 and modified HASBLED score was 1.5 ± 0.9. Thirty-one percent of rivaroxaban patients were initiated on the 15 mg dose, with the remainder prescribed 20 mg once daily. Using an estimated glomerular filtration rate cut-off of 50 mL/min/1.75m^2^, 6.4% of rivaroxaban patients were overdosed and 21.0% underdosed. Patients started on rivaroxaban were followed for an average of 1048 ± 693 days (2.9 years). Warfarin patients were followed for a mean 1,044 ± 727 days (2.9 years). Warfarin patients spent an average of 47 ± 28% (median: 50%) of their time in the target therapeutic INR range (linear interpolated assuming a target range of 2.0 to 3.0). Falsification analysis did not detect a difference between the two cohorts in the development of using urinary tract infection (HR = 0.97, 0.95–1.00).

**Fig. 2:**
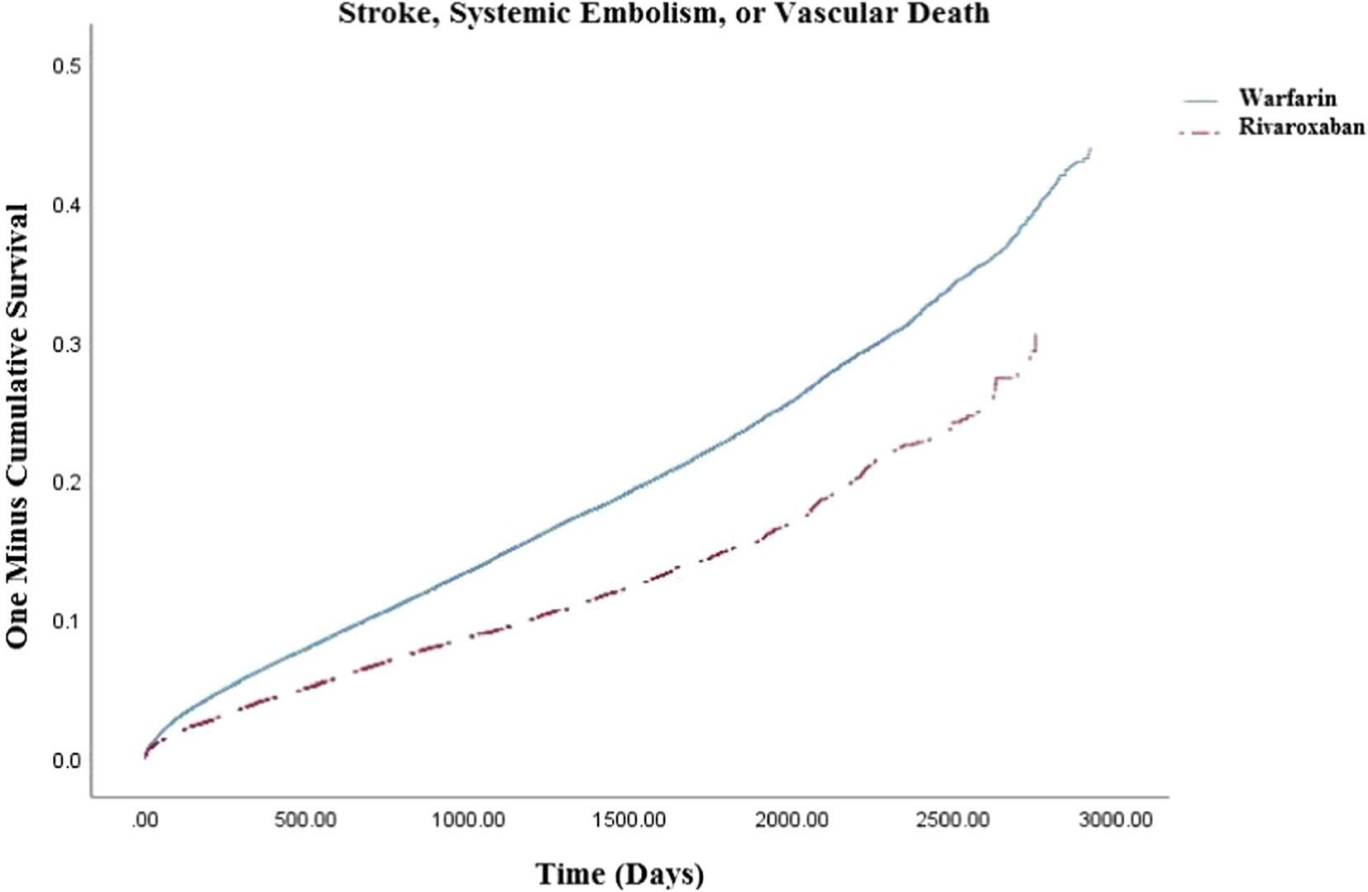
Kaplan–Meyer curve for primary effectiveness outcome

**Fig. 3:**
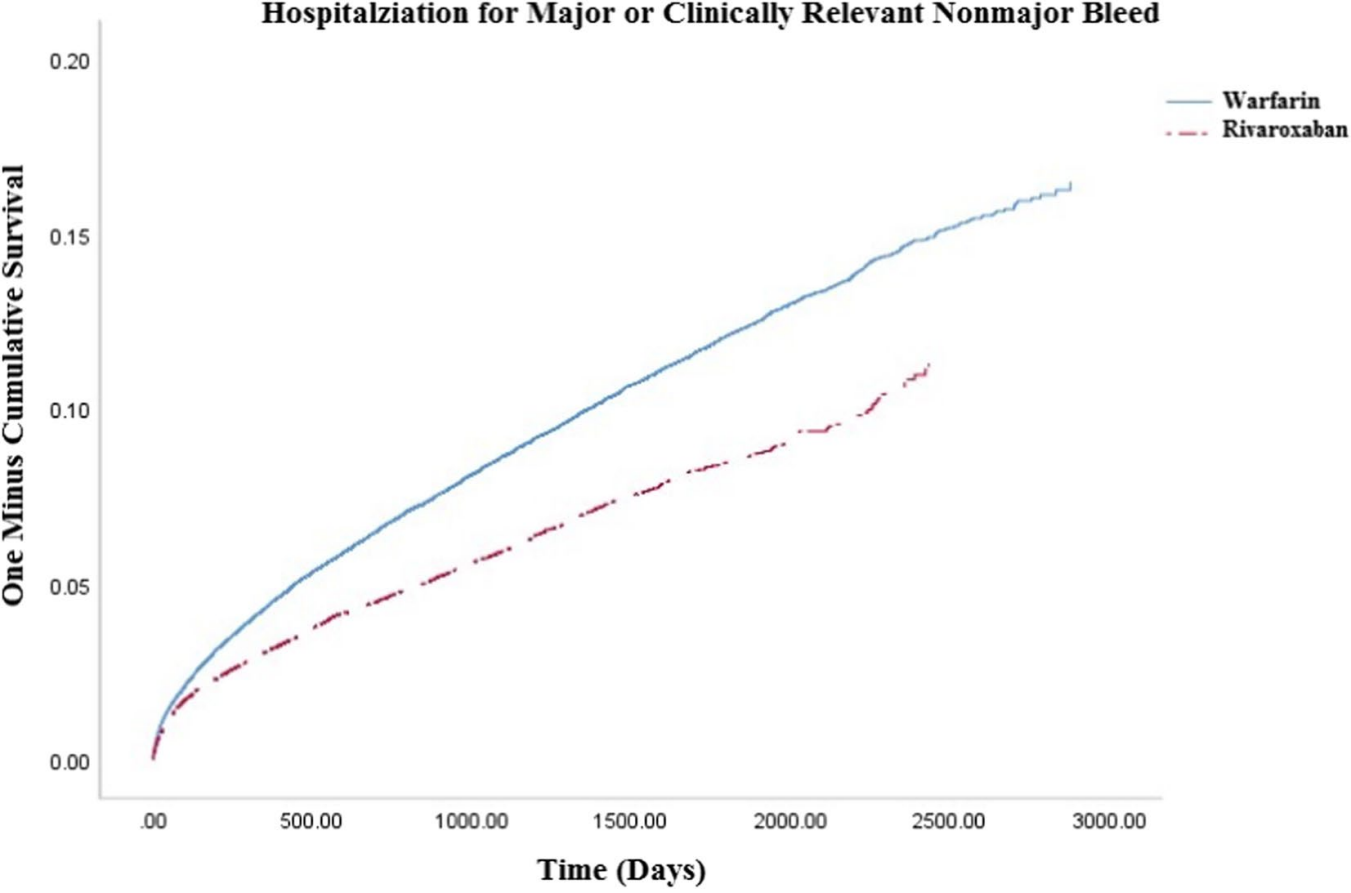
Kaplan–Meyer curve for primary safety outcome

Upon propensity score-overlap weighted Cox proportion hazard regression, we found rivaroxaban was associated with a reduced hazard of the composite outcome of SSE/vascular death (3.79 vs. 4.19; HR = 0.91, 95% CI 0.88–0.95) (Fig. 2) driven by a reduction in vascular death (2.81 vs. 3.18, HR = 0.90, 95% CI 0.86–0.95) (Table 2). When SSE was evaluated separately, no difference was detected (1.31 vs. 1.34; HR = 0.97, 95% CI 0.90–1.04). Hospitalization for any type of major/CRNM bleeding was less frequent in rivaroxaban users compared to warfarin users (2.17 vs. 2.31; HR = 0.94, 95% CI 0.89–0.99) (Fig. 3), as was critical organ bleeding (0.35 vs. 0.54; HR = 0.63, 95% CI 0.55–0.72) and intracranial hemorrhage (0.29 vs. 0.40; HR = 0.72, 95% CI 0.62–0.84) (Table 3). There was no difference in extracranial bleeding between rivaroxaban and warfarin (1.87 vs. 1.86; HR = 1.00, 95% CI 0.95–1.07), including gastrointestinal bleeding (1.50 vs. 1.42; HR = 1.06, 95% CI 0.99–1.13).

**Table 2:** Effectiveness outcomes

Outcome	Rivaroxaban N = 32,078 # of events (%/year)	Warfarin N = 83,971 # of events (%/year)	PS overlap weighted HR (95%CI)
Stroke, systemic embolism, vascular death	3497 (3.79)	10,077 (4.19)	0.91 (0.88–0.95)
Stroke, systemic embolism, myocardial infarction, vascular death	4074 (4.42)	11,420 (4.76)	0.94 (0.90–0.97)
Stroke or systemic embolism	1219 (1.31)	3275 (1.34)	0.97 (0.90–1.04)
Stroke, myocardial infarction, vascular death	4010 (4.34)	11,252 (4.69)	0.94 (0.90–0.97)
Ischemic stroke	1026 (1.10)	2519 (1.05)	1.05 (0.97–1.14)
Systemic embolism	128 (0.13)	420 (0.16)	0.82 (0.66–1.02)
Myocardial infarction	898 (0.99)	2267 (0.95)	1.04 (0.96–1.14)
Vascular death	2598 (2.81)	7641 (3.18)	0.90 (0.86–0.95)

*CI* confidence interval, *HR* hazard ratio, *PS* propensity score

**Table 3:** Bleeding outcomes

Outcome	Rivaroxaban N = 32,078 # of events (%/year)	Warfarin N = 83,971 # of events (%/year)	PS overlap weighted HR (95%CI)
Hospitalization for major or CRNM bleed	1989 (2.17)	5542 (2.31)	0.94 (0.89–0.99)
Major or CRNM bleed	6416 (6.95)	16,710 (6.95)	1.00 (0.97–1.03)
Major bleed	834 (0.90)	2687 (1.11)	0.80 (0.74–0.97)
Critical organ bleed	321 (0.35)	1344 (0.54)	0.63 (0.55–0.72)
Intracranial hemorrhage	257 (0.29)	1008 (0.40)	0.72 (0.62–0.84)
Extracranial bleed	1732 (1.87)	4450 (1.86)	1.00 (0.95–1.07)
CRNM bleed	5614 (6.09)	14,443 (6.00)	1.02 (0.98–1.05)

*CI* confidence interval, *CRNM* clinically relevant non-major, *HR* hazard ratio, *ICH* intracranial hemorrhage, *ISTH* International Society of Thrombosis and Haemostasis, *PS* propensity score

Exploratory analyses did not show a statistically significant interaction across most subgroups for either the SSE/vascular death or major/CRNM bleed outcomes (Table 4). One exception was the better relative effectiveness of rivaroxaban versus warfarin with the 20 mg rivaroxaban dose (compared to 15 mg) (p-interaction < 0.05). A second exception was the better relative effectiveness of warfarin versus rivaroxaban when the warfarin cohort was restricted to patients with a TTR ≥ 75% (11.6% of all warfarin users) during follow-up (p-interaction < 0.05). Use of alternative propensity score-based method to adjust for confounding or applying a 2-year follow-up cap did not impact the SSE/vascular death or major/CRNM bleed analysis results.

**Table 4:** Subgroup and sensitivity analysis

Subgroup	PS overlap weighted HR (95%CI)	
	SSE/vascular death	Major/CRNM bleeding
Age		
≥ 80 years	0.93 (0.87–1.00)	1.06 (0.96–1.18)
< 80 years	0.91 (0.86–0.96)	0.90 (0.84–0.96)
Sex		
Female	0.94 (0.88–1.01)	0.97 (0.89–1.06)
Male	0.89 (0.84–0.94)	0.92 (0.85–0.99)
eGFR		
> 50	0.93 (0.89–1.03)	0.92 (0.86–0.98)
30–50	0.89 (0.80–0.99)	1.03 (0.89–1.19)
< 30	0.79 (0.67–0.93)	1.02 (0.82–1.27)
Hemoglobin A1c		
≥ 8.5	0.86 (0.78–0.95)	0.89 (0.77–1.03)
< 8.5	0.93 (0.86–0.97)	0.95 (0.89–1.01)
Morbid obesity		
Yes	0.89 (0.82–0.99)	0.85 (0.75–0.95)
No	0.92 (0.87–0.96)	0.97 (0.91–1.03)
Heart failure		
Yes	0.92 (0.87–0.97)	1.02 (0.94–1.11)
No	0.89 (0.83–0.95)	0.87 (0.80–0.94)
Vascular disease		
Yes	0.91 (0.85–0.97)	1.03 (0.94–1.31)
No	0.89 (0.85–0.95)	0.89 (0.82–0.95)
Peripheral artery disease		
Yes	0.92 (0.85–1.00)	1.10 (0.98–1.23)
No	0.91 (0.86–0.95)	0.90 (0.84–0.96)
Revascularization		
Yes	0.94 (0.87–1.01)	0.99 (0.90–1.10)
No	0.88 (0.84–0.93)	0.97 (0.94–1.01)
Prior stroke		
Yes	1.02 (0.93–1.13)	0.98 (0.83–1.17)
No	0.89 (0.85–0.93)	0.93 (0.88–0.99)
Concomitant aspirin		
Yes	0.92 (0.88–1.01)	1.05 (0.97–1.30)
No	0.86 (0.84–0.93)	0.86 (0.80–0.93)
Frailty score		
Low	0.86 (0.80–0.94)	0.85 (0.77–0.94)
Moderate-to-high	0.92 (0.87–0.96)	0.99 (0.92–1.05)
Rivaroxaban dose		
20 mg	0.76 (0.72–0.80)*	0.86 (0.80–0.92)
15 mg	0.93 (0.88–0.99)	0.93 (0.86–1.02)
Warfarin time in therapeutic INR		
< 25%	0.72 (0.69–0.76)	0.64 (0.60–0.69)
25 to < 50%	0.72 (0.68–0.76)	0.74 (0.69–0.79)
50 to < 75%	1.02 (0.97–1.08)	1.03 (0.96–1.11)
≥ 75%	1.33 (1.22–1.44)	1.67 (1.48–1.85)
PS method		
OLW	0.91 (0.88–0.95)	0.94 (0.89–0.99)
sIPTW	0.94 (0.91–0.99)	1.00 (0.92–1.08)
1:1 PSM (caliper = 0.25 SD)	0.89 (0.85–0.94)	0.89 (0.83–0.95)
2-year follow-up cap	0.93 (0.88–0.98)	0.98 (0.92–1.06)

*CI* confidence interval, *eGFR* estimated glomerular filtration rate, *HR* hazard ratio, *INR* international normalized ratio, *OLW* overlap weighting, *PS* propensity score, *SD* standard deviation, *sIPTW* stabilized inverse probability weighting

*p-value for interaction < 0.05 after adjustment for multiple comparisons using the Benjamini, Hochberg and Yekutieli method to control for false discovery rates

## Discussion

In the present study we utilized detailed EHR data to evaluate > 116,000 patients with NVAF and comorbid T2D newly started on rivaroxaban or warfarin for a mean of ~ 2.9-years of follow-up. We found rivaroxaban use was associated with effectiveness and safety benefits versus warfarin; most notably, significant reductions in vascular death [10% relative risk reduction (RRR)], critical organ bleeding (37% RRR) and intracranial hemorrhage (28% RRR). These findings remained consistent across subgroups including baseline a1c level, with statistical interactions seen only when comparing the 20 mg versus 15 mg dosing subgroups for the SSE/vascular death outcome (an interaction based more on magnitude than direction of effect) and among patients with a well-controlled INRs (TTR ≥ 75%). Current findings also remained robust upon changes in confounding adjustment methodology employed and upon capping follow-up at a maximum of 2-years.

Our findings are generally consistent with those from the diabetes subanalysis of the Rivaroxaban Once-daily, Oral, Direct Factor Xa Inhibition Compared with Vitamin K Antagonism for Prevention of Stroke and Embolism Trial in Atrial Fibrillation (ROCKET AF Trial) [7, 8]. Bansilal and colleagues [8] evaluated0 5695 subjects with diabetes from ROCKET AF (mean CHADS_2_ score = 3.7 ± 1.0) and demonstrated rivaroxaban reduced the incidence rate of SSE/vascular death (4.23 vs. 5.17%/year, HR = 0.84 (0.70–1.00) and vascular death alone (2.83 vs. 3.65%/year, HR = 0.80, 95% CI 0.64–0.99). Of note, the vascular mortality reduction with rivaroxaban compared to warfarin in ROCKET AF was observed in diabetics but not in those without diabetes (HR = 1.08, 95% CI 0.89–1.30) (p-interaction = 0.037 for diabetic vs. non-diabetic subgroup comparison) [7, 8].

An administrative claims database study performed by Baker and colleagues [9] of nearly 24,000 patients provided confirmatory evidence to ROCKET AF [7, 8], suggesting rivaroxaban was at least as effective and safe as warfarin in NVAF patients with comorbid T2D. The investigators reported no statistically significant differences in ischemic stroke (HR = 0.83, 95% CI 0.59–1.17) or major bleeding (HR = 0.95, 95% CI 0.79–1.15) between the two inverse probability of treatment weighted (IPTW) OAC cohorts. Unfortunately, the IBM MarketScan claims data set utilized by the investigators does not provide mortality data, so vascular death could not be assessed [9]. This is noteworthy, since vascular death occurs in at least 7 out of 10 NVAF patients with diabetes [24] and appears to be the outcome most benefited by the preferential use of rivaroxaban in diabetics in ROCKET AF [7, 8] and in the present EHR study.

Another retrospective database study was performed by Chan et al. and investigated all DOACs versus warfarin in patients with comorbid NVAF and diabetes [25]. This study found no significant difference in SSE between DOACs and warfarin (HR = 0.89, 95% CI 0.79–1.02) but did find DOACs to be associated with a reduction in major bleeding (HR = 0.67, 95% CI 0.59–0.76). These findings are not inconsistent with those in our study, though our study importantly adds the outcome of vascular mortality. Our observed reduction in vascular mortality with rivaroxaban versus warfarin is bolstered by the findings of a meta-analysis performed by Patti and colleagues that demonstrated a reduction in vascular mortality with DOACs versus vitamin K antagonists in patients with comorbid NVAF and diabetes using data from four phase III RCTs (4.97 vs. 5.99%; relative reduction = 0.83, 95% CI 0.72–0.96) [26].

United States and European atrial fibrillation guidelines [1, 3] state that for stroke prevention, patients who are eligible for OAC should receive a DOAC in preference to a vitamin K antagonist (VKA) except in patients with mechanical heart valves or moderate-to-severe mitral stenosis (class 1A recommendations). European Society of Cardiology (ESC) and European Association for the Study of Diabetes (EASD) collaborative guidelines on the management of diabetes, pre-diabetes, and cardiovascular diseases additionally recommend (class 1A) DOACs over a VKA in patients with diabetes aged > 65 years with NVAF and a CHA_2_DS_2_VASc score ≥ 2, (if not otherwise contraindicated) [2]. Given vascular mortality is substantially increased in NVAF patients with comorbid T2D and the accumulating data suggesting DOACs [26] may be associated with up to a 17% relative and ~ 1% absolute risk reduction in vascular death, the practice of preferentially using DOACs over a VKA in a diabetic appears warranted [27].

Our study has limitations worth discussion. First due to the non-randomized, retrospective nature of this study, biases including misclassification, sampling, and confounding bias may impact internal validity [28]. We attempted to reduce the probability of misclassification bias by using validated coding schema [13–16, 20] and leveraging the wealth of laboratory and clinical observation data available in an EHR data set but not administrative claims databases [10, 28]. We used propensity score-overlap weighting to reduce the risk of confounding bias [18, 19]. While such propensity score-based methods serve to harmonize comparison groups with respect to patient characteristics, residual confounding cannot be ruled out [12]. Moreover, we performed falsification analysis which found, as anticipated, no difference between rivaroxaban and warfarin for the outcome of urinary tract infection. Second, due to the observational nature of this study, we did not have control over warfarin dosing or target INR chosen (though we assumed a target range of 2.0–3.0 for the purposes of TTR calculation). The TTR observed in our study (mean: 47%, median: 50%) was not dissimilar to that of warfarin patients enrolled in ROCKET AF (mean: 55%, median: 58%) [7] or to that observed in routine clinical practice (mean: 55%) [29]. Furthermore, we performed a subgroup analysis comparing rivaroxaban to warfarin patients stratified by TTR quartiles which suggested warfarin may be a good choice if patients can maintain a TTR ≥ 75%. Our data suggest only about 1 in every 10 warfarin patients can maintain that quality of INR control. This finding should also be interpreted with caution since data from the ORBIT registry suggest a past record of INR stability only weakly predicts future stability [30]. Third, time since diabetes diagnosis could not be accurately ascertained within the available data; and therefore, could not be included in the propensity score model. Fourth, cause of death was also not available in the database and therefore we used an algorithm consisting of hospitalization due to vascular cause within 365 days of death to identify “vascular” mortality. Notably, the vascular mortality rates observed in our study (rivaroxaban = 2.81%, warfarin = 3.18%) were similar to the vascular mortality rate in the diabetic sub-analysis of ROCKET AF (rivaroxaban = 2.83%, warfarin = 3.65%) [8]. Fifth, the EHR data set utilized for this study includes only US patients [10] making our findings most generalizable to a US population. Next, EHR data sets lack information on prescription medication claims [10]. Instead they provide data only on medications prescribed or self-reported (the latter is an advantage of EHRs as they allow for detection of over-the-counter medication use such as aspirin). The lack of prescription claims data makes ascertainment of OAC exposure (persistence and adherence) problematic. As a result, the present study only performed intent-to-treat (and not on-treatment) analyses. Finally, although Optum EHR data covers both insured and uninsured patients, it does not cover all institutions and therefore its possible follow-up events could be missed [10].

## Conclusion

In NVAF patients with T2D, rivaroxaban was associated with an ~ 10% RRR in vascular mortality and fewer bleeding-related hospitalizations versus warfarin, including a significant 37% RRR in critical organ bleeding and a 28% RRR in intracranial hemorrhage. Our data should provide clinicians with additional confidence in selecting rivaroxaban in NVAF patients with comorbid T2D.

## Data Availability

Data used in this study were obtained from Optum® under a license to Bayer AG (and provided to Dr. Coleman under a third-party agreement) and are not publicly available.

## References

[1] Hindricks G, Potpara T, Dagres N (2020). ESC guidelines for the diagnosis and management of atrial fibrillation developed in collaboration with the European Association of Cardio-Thoracic Surgery (EACTS). Eur Heart J.

[2] Grant PJ, Cosentino F (2019). The 2019 ESC Guidelines on diabetes, pre-diabetes, and cardiovascular diseases developed in collaboration with the EASD: new features and the ‘Ten Commandments’ of the 2019 Guidelines are discussed by Professor Peter J. Grant and Professor Francesco Cosentino, the Task Force chairmen. Eur Heart J.

[3] January CT, Wann LS, Calkins H (2019). 2019 AHA/ACC/HRS focused update of the 2014 AHA/ACC/HRS guideline for the management of patients with atrial fibrillation: a report of the American College of Cardiology/American Heart Association Task Force on Clinical Practice Guidelines and the Heart Rhythm Society. J Am Coll Cardiol.

[4] Xiong Z, Liu T, Tse G (2018). A machine learning aided systematic review and meta-analysis of the relative risk of atrial fibrillation in patients with diabetes mellitus. Front Physiol.

[5] Echouffo-Tcheugui JB, Shrader P, Thomas L (2017). Care patterns and outcomes in atrial fibrillation patients with and without diabetes: ORBIT-AF registry. J Am Coll Cardiol.

[6] Stroke Risk in Atrial Fibrillation Working Group (2007). Independent risk predictors of stroke in patients with atrial fibrillation: a systematic review. Neurology.

[7] Patel MR, Mahaffey KW, Garg J (2011). Rivaroxaban versus warfarin in nonvalvular atrial fibrillation. N Engl J Med.

[8] Bansilal S, Bloomgarden Z, Halperin JL (2015). Efficacy and safety of rivaroxaban in patients with diabetes and nonvalvular atrial fibrillation: the rivaroxaban once-daily, oral, direct factor Xa inhibition compared with vitamin K antagonism for prevention of stroke and embolism trial in atrial fibrillation (ROCKET AF trial). Am Heart J.

[9] Baker WL, Beyer-Westendorf J, Bunz TJ (2019). Effectiveness and safety of rivaroxaban and warfarin for prevention of major adverse cardiovascular or limb events in patients with non-valvular atrial fibrillation and type 2 diabetes. Diabetes Obes Metab.

[10] Optum. Optum EHR offering. Optum Inc. 2018. https://www.optum.com/campaign/ls/data-new-era-of-visibility/download.html. Accessed 18 Dec 2020.

[11] Khokhar B, Jette N, Metcalfe A (2016). Systematic review of validated case definitions for diabetes in ICD-9-coded and ICD-10-coded data in adult populations. BMJ Open.

[12] Austin PC (2011). An introduction to propensity score methods for reducing the effects of confounding in observational studies. Multivar Behav Res.

[13] Centers for Medicare and Medicaid Services. Chronic conditions data warehouse. https://www2.ccwdata.org/web/guest/home/. Accessed 18 Dec 2020.

[14] Elixhauser A, Steiner C, Harris DR (1998). Comorbidity measures for use with administrative data. Med Care.

[15] Quan H, Li B, Couris CM (2011). Updating and validating the Charlson comorbidity index and score for risk adjustment in hospital discharge abstracts using data from 6 countries. Am J Epidemiol.

[16] Gilbert T, Neuburger J, Kraindler J (2018). Development and validation of a Hospital Frailty Risk Score focusing on older people in acute care settings using electronic hospital records: an observational study. Lancet.

[17] Sterne JA, White IR, Carlin JB (2009). Multiple imputation for missing data in epidemiological and clinical research: potential and pitfalls. BMJ.

[18] Thomas LE, Li F, Pencina MJ (2020). Overlap weighting: a propensity score method that mimics attributes of a randomized clinical trial. JAMA.

[19] Li F, Thomas LE, Li F (2019). Addressing extreme propensity scores via the overlap weights. Am J Epidemiol.

[20] Cunningham A, Stein CM, Chung CP (2011). An automated database case definition for serious bleeding related to oral anticoagulant use. Pharmacoepidemiol Drug Saf.

[21] Schulman S, Kearon C on behalf of the subcommittee on control of anticoagulation of the Scientific and Standardization committee of the International Society on Thrombosis and Haemostasis (2005). Definition of major bleeding in clinical investigations of antihemostatic medicinal products in non-surgical patients. Scientific and Standardization Committee Communication. J Thromb Haemost.

[22] Benjamini Y, Yekutieli D (2001). The control of the false discovery rate in multiple testing under dependency. Ann Stat.

[23] Langan SM, Schmidt SA, Wing K (2018). The reporting of studies conducted using observational routinely collected health data statement for pharmacoepidemiology (RECORD-PE). BMJ.

[24] Pokorney SD, Piccini JP, Stevens SR (2016). Cause of death and predictors of all-cause mortality in anticoagulated patients with nonvalvular atrial fibrillation: data from ROCKET AF. J Am Heart Assoc.

[25] Chan Y, Lee H, Ru P (2020). Effectiveness, safety, and major adverse limb events in atrial fibrillation patients with concomitant diabetes mellitus treated with non-vitamin K antagonist oral anticoagulants. Cardiovasc Diabetol.

[26] Patti G, Di Gioia G, Cavallari I (2017). Safety and efficacy of nonvitamin K antagonist oral anticoagulants versus warfarin in diabetic patients with atrial fibrillation: a study-level meta-analysis of phase III randomized trials. Diabetes Metab Res Rev.

[27] Yamagishi S (2019). Concerns about clinical efficacy and safety of warfarin in diabetic patients with atrial fibrillation. Cardiovasc Diabetol.

[28] Gandhi SK, Salmon W, Kong SX (1999). Administrative databases and outcomes assessment: an overview of issues and potential utility. J Manag Caerr Spec Pharm.

[29] Baker WL, Cios DA, Sander SD, Coleman CI (2009). Meta-analysis to assess the quality of warfarin control in atrial fibrillation patients in the United States. J Manag Care Pharm.

[30] Pokorney S, Simon DN, Thomas L (2015). The myth of the stable INR patient: results from ORBIT-AF. J Am Coll Cardiol.

